# The exercise metabolome: acute aerobic and anaerobic signatures

**DOI:** 10.1080/15502783.2022.2115858

**Published:** 2022-10-11

**Authors:** Joseph K. Pellegrino, Tracy G Anthony, Peter Gillies, Shawn M. Arent

**Affiliations:** a Department of Health and Human Performance, University of Scranton, Scranton, PA, USA; b Department of Nutritional Sciences, Rutgers University, New Brunswick, NJ, USA; c Department of Exercise Sciences, University of South Carolina, Columbia, SC, USA

**Keywords:** Metabolomics, exercise, Anaerobic, Aerobic

## Abstract

**Background:**

Exercise modality differentially alters body composition and physical performance. Metabolic changes underlying these outcomes can be tracked through assessment of circulating metabolites. Here, global responses to an acute bout of aerobic or anaerobic exercise were compared in the serum of male and female subjects using a discovery-based metabolomics platform.

**Methods:**

On separate days, 40 healthy, active participants completed 45 min of aerobic cycling or resistance exercise, and blood samples were collected at rest, immediately after (T1) and 1 hour post-exercise (T2) to examine the serum metabolomic landscape.

**Results:**

The two exercise metabolomes appeared more similar than different in this healthy cohort. Overall, metabolomic signatures of both exercise modalities were markedly altered from rest at T1, and returned toward baseline by T2. Metabolomic perturbations at T1 and the T1-T2 rate of recovery post-exercise were greater following aerobic cycling than resistance exercise. Shared signatures included elevations in purine metabolism, substrate catabolism and mobilization, and inflammatory signaling. Aerobic exercise resulted in greater substrate diversity and use of fatty acids, whereas resistance exercise displayed higher purine turnover and glycolytic flux.

**Discussion:**

Individual metabolite differences between conditions were seen in magnitude but not direction. Metabolomic signatures of the exercise responses appeared fairly robust across exercise modalities. An initial perturbation and subsequent shift toward recovery by an hour post-exercise defined the signature in our healthy cohort. The expedited recovery following aerobic cycling may be explained by globally elevated lipid metabolism.

## 1. Introduction

A rich body of literature supports the salutary effects of exercise. Regular physical activity correlates with lowered risk of chronic diseases and reduced mortality rates [1]. The utility of exercise for prevention, management, and treatment of various diseases is well documented, particularly those related to metabolic disorders and cardiovascular conditions [2–4]. Despite this knowledge, there is still debate surrounding the proper prescription of exercise to improve health status.

Because of the shear complexity of exercise’s multi-systemic response, knowledge gaps in our understanding are hard to fill. Global serum metabolomic profiling provides a broad platform from which to characterize the exercise response and assess whether circulating metabolic signatures can be used to differentiate between various exercises. A study characterizing the metabolomic response to multiple stressors, including exercise, was able to differentiate between these challenges using the resultant metabolomic profiles [5]. These findings indicate each stressor produces a unique metabolomic signature, if the platform is sensitive enough to map it.

Previously published metabolomic explorations of the response to exercise in humans found the greatest disruption to the resting metabolome to occur immediately after exercise, with individual metabolites having various post-exercise response patterns throughout the following 24 hours [5–8]. Major changes to the metabolome brought on through aerobic exercise point to energy metabolism: adenine, tricarboxylic acid cycle intermediates, and mobilization and catabolism of substrates (carbohydrates, lipids, amino acids, and ketones) and the antioxidant response [5–14]. Metabolic signatures have been related to improvements or changes in cardiovascular function [4], insulin sensitivity [7], immunity [9,10], and calcium homeostasis [10]. Studies of anaerobic exercise have also identified energy balance and oxidative stress as perturbed pathways [15,16]. Enhanced glycolysis and emphasized branched-chain amino acid metabolism are reported following anaerobic exercise as well [16,17].

Aerobic and anaerobic exercises are conceptually different from one another. Anaerobic exercise typically emphasizes neuromuscular strength gains and muscle hypertrophy while aerobic exercise primarily targets cardiorespiratory fitness and mitochondrial biogenesis [18,19]. Still, there are overlapping benefits to each as well. Both modalities foster improved health, body composition, and energy levels, plus recent findings support cardiovascular benefits with high-intensity interval training [19,20]. Indeed, because of the myriad benefits seen with so many different exercise modalities, questions persist surrounding exercise prescription for optimal health and fitness. Characterization of the acute metabolomic responses to different exercise modes will provide insight into how to prescribe aerobic and anaerobic exercises.

The aim of the present study was to use an exploratory metabolomics platform to examine the response pattern of active, healthy individuals to acute bouts of aerobic versus anaerobic exercise. We hypothesized that the two bouts would partially overlap in their responses, representing the general effects of exercise, but also include subsets of differing metabolites, delineating the unique effects of each type of exercise.

## 2. Methods

### 2.1. Research design

All procedures were approved by the Rutgers University Institutional Review Board (15-417Mx), and participants provided written informed consent prior to enrollment. Active, healthy young adults were exposed to two different exercise bouts, aerobic cycling (AC) and anaerobic resistance exercise (RE). Metabolomic profiles were characterized at rest (T0), immediately after (T1) and 1 hour post-exercise (T2). This design was implemented to characterize and differentiate between the acute responses to aerobic and anaerobic exercise in healthy individuals. Active participants were targeted as research has shown more heterogeneity in the metabolome of sedentary individuals [8]. All testing and conditions were held in the Rutgers University Center for Health and Human Performance.

### 2.2. Participants

Forty (20 males + 20 females) individuals (age: 24 ± 3.1 years, height: 168.8 ± 9.3 cm, weight: 67.2 ± 9.4 kg) were recruited for participation. Inclusion criteria were the absence of any known cardiorespiratory, metabolic, or other health issues and a minimum of 2 years of regular participation in their current physical activity regimen. Participants were excluded if they suffered illness or injury that altered the training frequency, intensity, or volume for a period of ≥ 1 week within the past six months, or if they used tobacco products or recreational drugs. To avoid any metabolic adaptations to supplement use or discontinuance, participants were asked to maintain use of any supplements they had been using for at least the prior six months and to refrain from beginning use of any new supplements during the study. They were also asked to avoid the use of NASAIDs throughout the study. In the event that the participants had recently (<6 months) started the use of a supplement prior to the study, they would undergo a 4-week washout prior to enrollment. However, this wound up not being necessary for any of the participants. Participants reported to the lab for two testing trials followed by two experimental conditions randomized for order. Testing and experimental sessions were conducted for 3-7 days following the previous session.

## 3. Exercise testing trials

### 3.1. Visit 1

Participants reported to the lab following a 2-hour fast and having refrained from exercise for the previous 24 hours. Upon arrival, body composition testing with a Bod Pod (Cosmed, Concord, CA) was performed using the Brozek equation [21]. Following body composition assessment, a 10-minute warm-up was permitted before completing a continuous graded exercise test on a Velotron cycling ergometer (Racermate, Seattle, WA). Depending on the estimated work-capacity, individuals started between 70 and 220 Watts (W), increasing wattage by 30 W every third minute. For testing, participants were fitted with a Polar M-400 heart rate (HR) monitor (Polar Inc., Bethpage, NY) and Quark C-PET metabolic measuring system (Cosmed, Concord, CA) for HR and breath-by-breath gas analysis using 15-second rolling averages. All participants continued until volitional fatigue or the inability to keep cadence ≥ 80 rpm and met the following criteria for attainment of VO_2MAX_: plateau in VO_2_ with increasing workload, HR within 10 bpm of age-predicted max-HR, respiratory exchange ratio ≥ 1.1 [22].

Maximal aerobic power output (PO_MAX_) was calculated as [23]:

$PO_{MAX}=\;Wattage\;of\;last\;completed\;stage+\;30\;\times \;\frac{seconds\;\;into\;\;final\;\;stage}{180}$

Seventy percent of this wattage was used as the intensity prescription during AC. Pilot testing showed this intensity to provide an arduous exercise bout within the 45-minute duration used for exercise conditions.

### 3.2. Visit 2

During the second visit to the lab, participants completed 10-repetition max (10RM) testing, following National Strength and Conditioning Association guidelines [24,25], for seven lower-body exercises. Determination of 10RM took three sets on average. In order, exercises were back squat, leg press, Romanian deadlift, lunges, prone leg curls, leg extensions, and seated calf raises. A certified strength and conditioning specialist administered testing to ensure participant safety and proper execution of all lifts. The same seven exercises, in the same order, were performed during RE.

## 4. Exercise conditions

### 4.1. Control factors

In accordance with findings on variability in metabolomics, precautions were taken to minimize extraneous infuence [26,27,28]. Individuals refrained from any exercise 24 hours before and arduous exercise 48 hours prior to either condition. Participants were provided a 24-hour food log so that diet could be replicated for the second condition. They were encouraged to eat as normal, with the only instruction being to consume a meal 2–3 hours prior to the visit, with only water ingested after this point. The exact time of day was matched between conditions for each participant, and all exercise sessions started between 8:00 AM and 12:00 PM. Finally, individuals were instructed to avoid caffeine that morning. Exercise duration was 45 minutes for both AC and RE, and order was randomized for all 40 participants.

### 4.2. Aerobic session

Upon arrival, participants sat quietly for 10–15 minutes and the T0 blood draw was performed with venipuncture of the antecubital vein. This was followed by a 15-minute warm-up period before commencing with the prescribed exercise. The AC bout was a 45-minute steady-state ride at 70% PO_MAX_ on the same ergometer as used during VO_2MAX_ testing. A researcher monitored HR and cadence during the session and participants were encouraged to keep their cadence between 90 and 100 rpm. In order to maintain an aerobic effort, if HR went above 90% of the HR_MAX_ value observed during VO_2MAX_ testing or cadence below 80 rpm, wattage was adjusted until HR and/or cadence normalized within the desired range. RPE was collected using a modified Borg scale immediately after exercise

### 4.3. Anaerobic session

RE was identical in structure to AC, including all aspects of sample collection. Only, the cycling bout was replaced with resistance training using the seven exercises listed above. Three sets of 10 repetitions were performed for each exercise with a 90-second recovery between all sets. This combination of load, rest interval, sets, and repetition counts falls within the guidelines for hypertrophy training, qualifying as an ‘anaerobic’ working intensity. A Certified Strength & Conditioning Specialist oversaw all sessions to ensure participant safety and proper execution of all lifts. If the workload was too high (participant could not complete a full set of 10), they were spot-assisted to completion and weight was adjusted accordingly on any subsequent sets of that exercise. All RE bouts took between 44 and 47 minutes.

### 4.4. Metabolomics analysis

Upon cessation of each exercise condition, participants were promptly escorted into an adjacent phlebotomy room where an indwelling catheter was inserted into the contralateral arm for subsequent draws, T1 and T2. To prevent coagulation, the line was flushed with 2 ml saline immediately after and every 15 minutes between draws. Nutrient intake was not permitted at any point during the session, but participants were allowed to drink water *ad libitum*.

Following collection and processing, serum samples were aliquoted into 2.5-ml cryovials and stored at −80°C until all testing was complete (up to 3½-months). Samples were packed on dry ice and shipped to the Metabolon® core facility in Durham, NC for analysis with Metabolon’s Discovery Panel of 754 metabolites. Automated ultra-high-performance liquid chromatography/electrospray ionization tandem mass spectrometry was used for metabolite identification. Raw data were extracted, peak identified, quality-control processed with internal and external controls, and quantified by area-under-the-curve using Metabolon’s hardware and software. Compounds were then identified by comparison to library entries of purified standards or recurrent unknown entities based on three criteria: retention index, mass, and the MS/MS forward and reverse scores between the experimental data and authentic standards. All samples were run in a single batch to reduce variance.

Values were normalized to raw area counts through log-transformation and rescaled to set the median equal to 1.00 for each metabolite. Any missing values were imputed with the minimum value. Statistical procedures were carried out using these values. All data were re-run excluding any metabolites using > 25% imputed values. This exclusion did not impact global biochemical profiles, and inclusive data are reported herein.

### 4.5. Statistical procedures & data analysis

Unsupervised approaches to analysis were performed in Array Studio and included hierarchical clustering and principal component analysis (PCA). The hierarchical clustering method performed was complete clustering using the Euclidean distance, where each value was factored independently as a vector to all other metabolite values. Condition clusters were identified and superimposed on the output. PCA by singular value decomposition was used to reduce dimensionality and generate data clusters for all samples showing differences between condition and time.

Metabolites for each condition were separately entered into Metaboanalyst (http://www.metaboanalyst.ca) for pathway analysis. Pathway impact and significance were calculated based on the *Homo sapiens* Kegg Pathway relations. Algorithms included the Hypergeometric Test for over representation analysis and Relative-betweenness Centrality for pathway topology analysis. Pathway analysis highlighted potential metabolic areas of overlap and distinction. A false discovery rate (FDR)-corrected p-value < 0.05 and impact factor > 0.1 were used to determine significantly altered pathways.

A 2 × 3 (condition × time) Repeated Measures Analysis of Variance (RM-ANOVA) with follow-up univariate ANOVAs for delta values between time points were run for all metabolites using a p-value < 0.05. Due to multiple comparisons in metabolomics data sets, the Benjamin-Hochberg (FDR) was determined with a stringency set at a q-value of 0.1. For values determined to be statistically significant, by both p and q criterions, magnitude of change was used to further identify meaningful metabolite changes. Average changes from T0-T1 to T0-T2 for each exercise were calculated and considered physiologically significant when a 2-fold difference was reached between the resting and post-exercise values. With resting values set at 1.00, this meant a post-exercise value ≥ 2.00 or ≤ 0.50 [29]. Unless otherwise stated, all data discussed below meet all three criteria outlined above. Values from the RM-ANOVA analyses are given as fold-change values in arbitrary units, equivalent to expression as a percentage of resting level.

## 5. Results

### 5.1. Participant characteristics

Fitness characteristics showed a healthy, fit group of active individuals (Table 1). Male participants were significantly taller, heavier, and leaner than their female counterparts. Moreover, fitness measures reflected greater strength (squat and leg press 10RM-weight) and aerobic power (VO_2MAX_ and PO_MAX_) but not VT for males than females in absolute terms. However, differences between male and female groupings were found to be largely anthropomorphic, as strength and endurance differences were eliminated when scaled to fat-free mass.

**Table 1.** Descriptive characteristics of individuals in the study.

Category	Mean ± SD	Males (20)	Females (20)
Age (years)	23.0 ± 3.1	25.0 ± 3.9	23.1 ± 2.3
Height (cm)*	168.8 ± 9.3	175.2 ± 7.3	162.5 ± 6.2
Mass (kg)*	67.2 ± 9.4	72.9 ± 7.8	61.5 ± 7.2
% Body Fat*	18.1 ± 7.5%	12.8 ± 5.7%	23.3 ± 4.8%
Fat Free Mass (kg)*	55.2 ± 9.7	63.3 ± 4.9	47.1 ± 5.8
relative VO2MAX (ml/kg/min)*^†^	46.5 ± 8.0	49.6 ± 10.1	43.3 ± 3.2
VT (% of VO2MAX)	72 ± 11%	73 ± 14%	71 ± 0.1%
POMAX (Watts)*^†^	218 ± 57	257 ± 53	178 ± 23
Squat 10RM (kg)*^†^	72 ± 29	88 ± 32	57 ± 17
Leg Press 10RM (kg)*^†^	182 ± 62	212 ± 65	153 ± 42

SD = standard deviation; FFM = fat free mass, VO_2MAX_ = max aerobic capacity; PO_MAX_ = max aerobic power; 10RM = 10-repetition max weight.

* Males significantly different than females, p < 0.05

^†^Difference eliminated when scaled to FFM

### 5.2. Session characteristics

After adjustments, the average wattage used during AC was 69.8 ± 0.8% PO_MAX_, and the average weight used across lifts was 89.9 ± .6% of 10RM during RE. Ratings of perceived exertion (1–10 scale) at the conclusion of each session were equivalent (9.1 ± 0.7 for AC, and 9.2 ± 0.9 for RE, p > 0.05), indicating both sessions were perceived as equally challenging for the participants.

### 5.3. Metabolomic signatures

#### 5.2.1. Resting comparison

Comparison of resting values between visits revealed no detectable day-to-day variation between conditions for any of the metabolites on the panel (p > 0.05, q > 0.1 for all 754 metabolites at T0).

#### 5.2.2. Common post-exercise response

**Figure 1.**
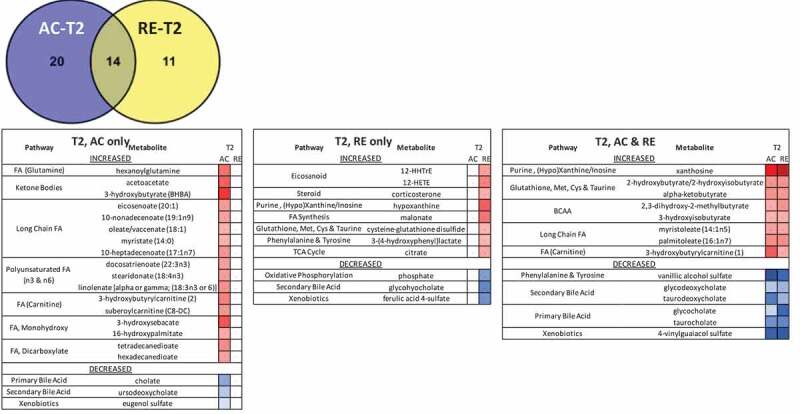
(Continued)
**Figure 2.**
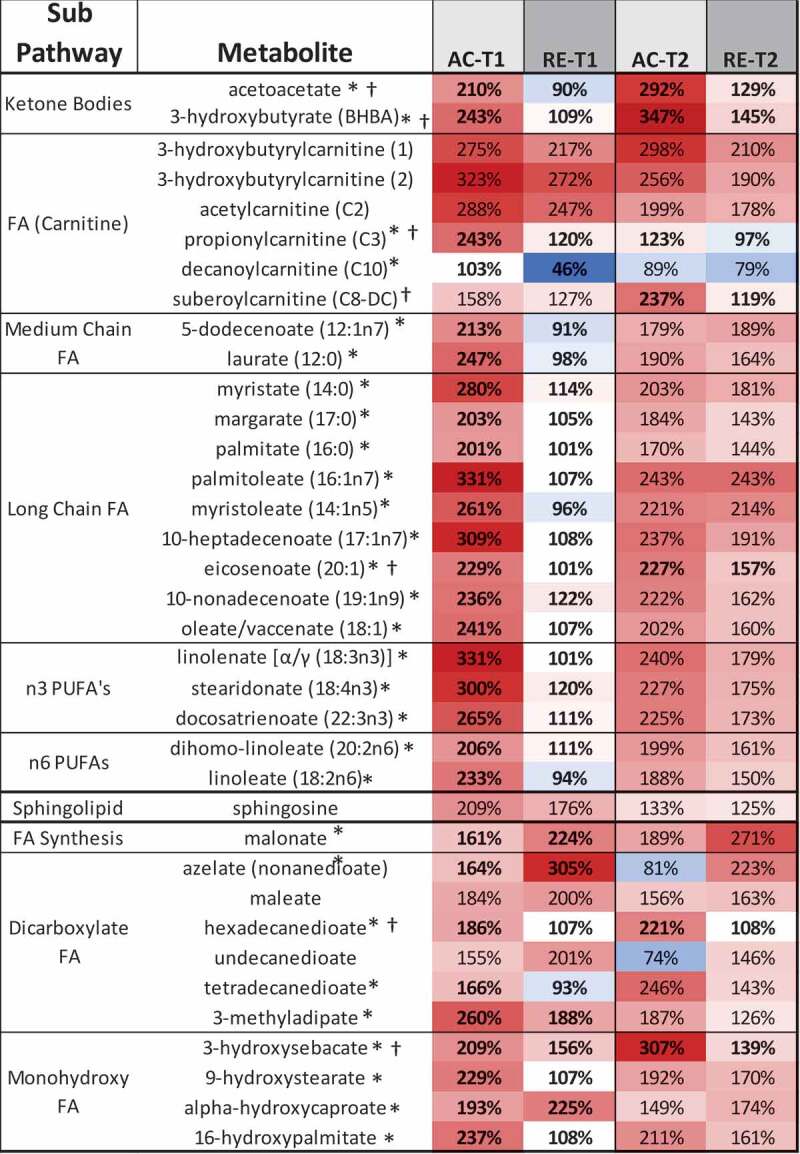
Heatmaps, with numeric data for significantly altered metabolites. Values expressed as a percentage of resting level. Red = increase, blue = decrease from baseline. Significant changes in bold. **A)** FA metabolites in response to AC and RE. Substantially more elevation in FA metabolism in response to AC was seen at both time points. Additionally, FA metabolites showing a response at RE-T1 tended to be markers of distressed or inadequate mitochondrial FA processing (malonate and most dicarboxylate- and monohydroxy-FA’s). A split in PUFA metabolism between n3 and n6 metabolites following exercise was evident. FA metabolites with antioxidant properties (3-methyladipate and 16-hydroxypalmitate) were greater in AC > RE. Values in **bold** are significantly different across exercises at that time point. **B)** Heat map of TCAC intermediates & β-citrylglutamate levels immediately (T1) and 1-hour (T2) post-exercise. Values expressed as a % of resting level at each time point. **C)** Fatty-acylcholines & phospholipids significantly (p < 0.05, q < 0.1) altered > 2-fold. Values expressed as % of resting level at each time point. Significant changes are shaded blue -darker = greater magnitude of change. AC = aerobic exercise; RE = anaerobic exercise; T1 = immediate post = exercise; T2 = 60-minutes post-exercise. **Bold** indicates a significant change from baseline, p < 0.05 & q < 0.1. **D)** Outline of major metabolic pathways activated by exercise. AC elicited greater lipid metabolism due to greater FA substrate selection and a metabolomic profile indicating more membrane turnover and downstream lipid mediator signaling. RE elicited greater anaerobic glycolysis and purine salvage due to a greater demand on rate of energy production. This highlights the fundamental difference between AC and RE as volume- versus intensity-dependent stressors, respectively.

Overlaps in the responses to AC and RE were apparent at both time points. Twenty-nine metabolites at T1 and 14 at T2 were similarly altered by both exercises (Figure 1). T1 metabolites indicated increased purine salvage, glycolytic flux, tricarboxylic acid cycle throughput, ketone and fatty acid (FA) metabolism, glutathione processing, inflammatory activity, and reduced acylcholines and 2° bile acids. Purine turnover, FA/ketone metabolism, and glutathione cycling were still evident at T2 (Figures 1 and 2). No metabolites changed in opposing directions for AC and RE at either time point.
**Figure 1.**
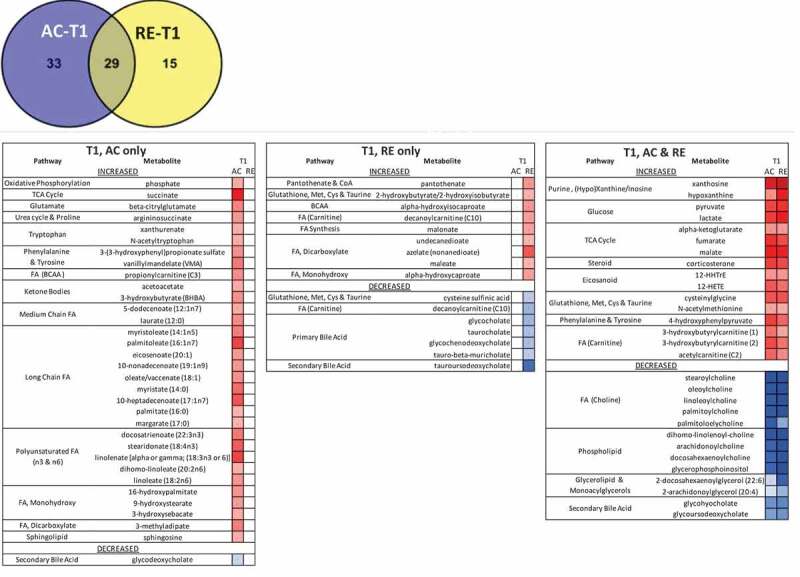
Comparison of responses to AC and RE at T1 (A) and T2 (B). Venn diagrams indicate number of metabolites significantly altered by each exercise from baseline value at that time. Heat map includes listing of metabolites corresponding to Venn diagrams comparing AC to RE for the labeled time and exercise.

Red: increase from baselineBlue: decrease from baselineAC: aerobic exercise session; RE = anaerobic exercise session; T0: resting T1: immediate post-exercise; T2: 60-minute post-exercise.

*AC significantly different from RE at T1

**Figure 2.**
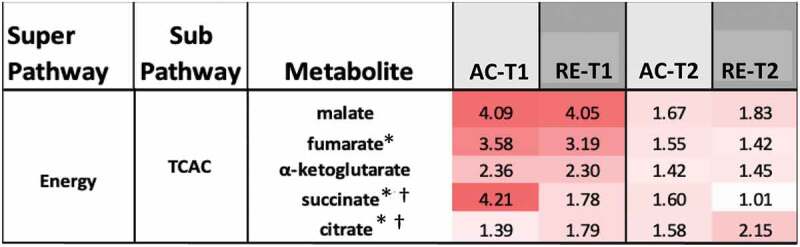
(Continued)

**Figure 2.**
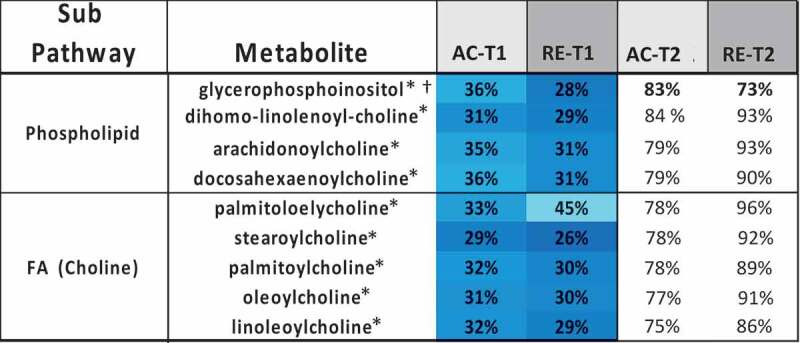
(Continued)

**Figure 2.**
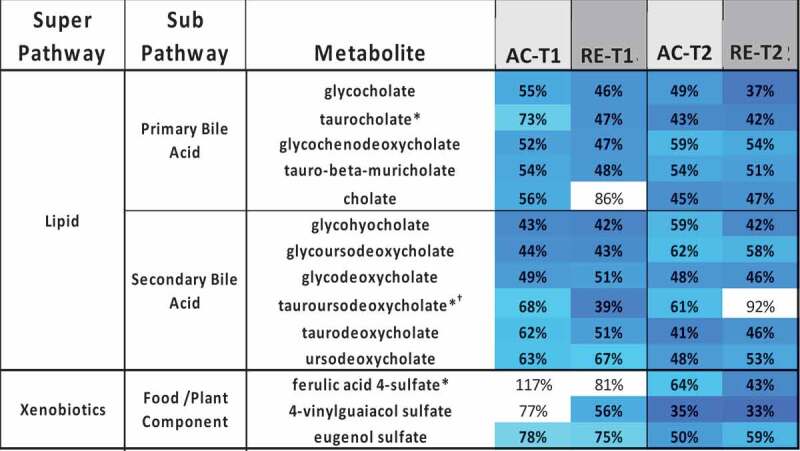
(Continued)

**Figure 2.**
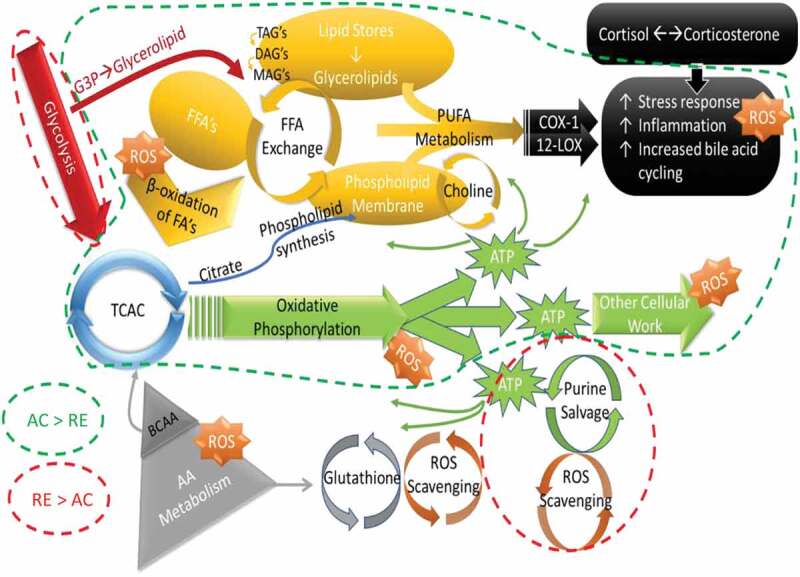
(Continued)

Unsupervised PCA of the data by session, participant, and time revealed distinct clustering patterns (Supplementary Figure 1A). Resting data for AC and RE showed no separation between sessions. Post-exercise, data were displaced from T0, with a greater shift at T1, demonstrating a disruption to the resting metabolome immediately post-exercise followed by a recovery within 1 hour for both conditions. Hierarchical clustering of individual metabolites across time points yielded mixed groupings from AC and RE (Supplementary Figure 1B), suggesting no clear delineation between exercise modes.

Pathway analysis of changes between T0 and T1 (during exercise) gave highly similar responses between exercises, mostly involving energy metabolism: tricarboxylic acid cycle, butyric acid metabolism, and glycolysis. AC, however, had consistently larger impact factors in most pathways than did RE. Moreover, AC resulted in greater fat metabolism, while RE included larger impact factors for xanthine metabolism and glycolysis (Supplementary Figure 1C-F). Stress signaling was highlighted as well, with increased adrenal (catecholamines and glucocorticoids), antioxidation (glutathione cycling), and inflammatory activity including the eicosanoids and other lipid mediators.

#### 5.2.3. Unique AC vs RE responses

Despite an overarching similarity between metabolomic signatures for AC and RE, some differences were evident. Separation between exercises was greater at T1 than T2. Although the metabolomic shift was greater at T1 than T2 for both exercises, this pattern was more evident for AC, where a tighter clustering of data was observed. Several markers even increased from T1 to T2 following RE, FA metabolites in particular. AC also showed a more prolific response in terms of number of metabolites as well as mean fold-change (Figures 1, 2**, Supplementary Figure 2**). Notably, 25 of the 33 metabolites altered for AC-only at T1 related to FA metabolism, including 17 actual FAs (Figure 1). While there were a number of elevated short-chain FAs in response to both exercises, the AC response included numerous metabolites indicating aerobic mitochondrial oxidation. Nearly every long-chain FA, as well as most of the medium-chain FA, polyunsaturated FA (PUFA), and both ketone bodies on the panel were elevated immediately following AC only (Figure 2(a)). By contrast, there was evidence of comparatively less mitochondrial metabolism and greater lysosomal FA oxidation at RE-T1, indicated by increased malonate, azelate, and maleate (Figure 2(a)). RE did, however, increase aerobic FA metabolism by T2, whereas, though FA metabolites remained elevated at AC-T2 they largely decreased T1-T2 following AC (Figure 2(a)).

**Figure 3.**
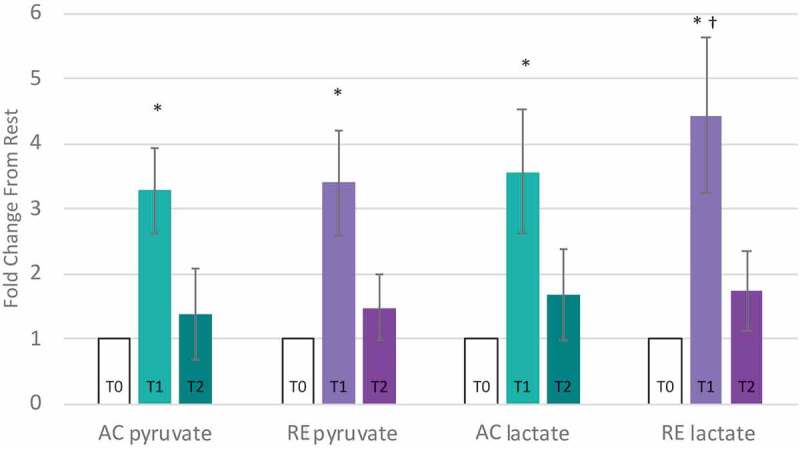
Glucose Metabolism is higher at T1 for RE. (T1) for aerobic (AC) and anaerobic exercise (RE). After 1-hour of recovery (T2) the pro-inflammatory metabolites returned to baseline following AC, but remained elevated following RE.

**Figure 4.**
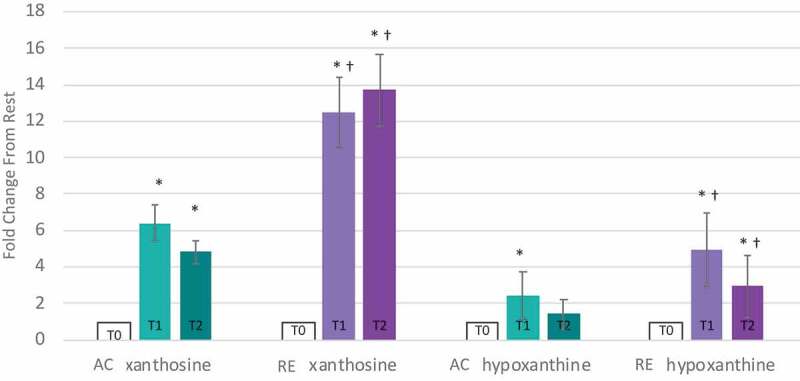
Responses of purine salvage markers (xanthosine & hypoxanthine) to aerobic (AC) and anaerobic exercise (RE) expressed as fold-of change form resting value. T0: resting, T1: immediately post-exercise, T2: 1-hour post-exercise.

Evidence of a comparatively limited FA metabolism in response to RE at T1 was offset through other bioenergetic pathways. Lactate and pyruvate were elevated by AC and RE T1, yet the rise in lactate was 31% greater in response to RE than AC (p < 0.05, Figure 3), indicating greater anaerobic glycolysis during RE. Further, citrate was > 2-fold elevated at T2 in response to RE only, while other tricarboxylic acid cycle intermediates responded similarly to each exercise (Figure 2(b)). Moreover, despite directional similarities, magnitude of purine metabolism differed between conditions. Increases in xanthosine and hypoxanthine were roughly 2-fold greater for RE than AC at T1. While xanthosine started to decline in the hour following AC, it continued to increase following RE, and hypoxanthine was significantly elevated at T2 for RE only (Figure 4). Notably, xanthosine was the only metabolite on the panel to be significantly altered more than 2-fold following both exercises at both time points.

* Significantly different from resting (T0).^†^ Significant difference between AC and RE, p < 0.05, q < 0.1, magnitude > 2-fold.*AC: aerobic exercise session; RE = anaerobic exercise session; T0: resting T1: immediate post-exercise; T2: 60-minute post-exercise.*

* Significant change from resting value, p < 0.05, q < 0.1, magnitude > 2-fold.^†^ Significant difference between AC and RE, p < 0.05, q < 0.1, magnitude > 2-fold.AC: aerobic exercise session; RE = anaerobic exercise session; T0: resting T1: immediate post-exercise; T2: 60-minute post-exercise.

**Figure 5.**
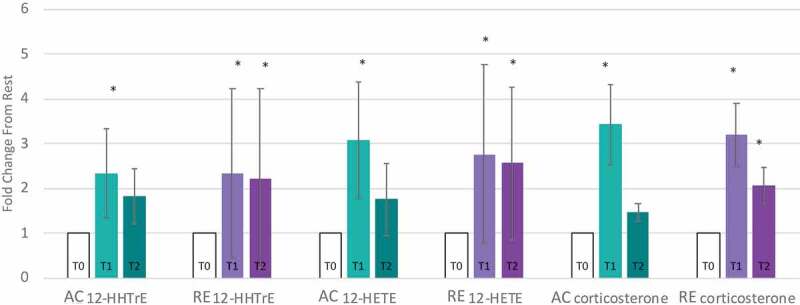
Pro-inflammatory markers are elevated immediately following exercise (T1) for aerobic (AC) and anaerobic exercise (RE). After 1-hour of recovery (T2) the pro-inflammatory metabolites returned to baseline following AC, but remained elevated following RE.

Numerous metabolites reflecting the stress response followed different patterns for AC and RE. Vanillylmandelate, a breakdown product of the catecholamine epinephrine, was 2-fold elevated at AC-T1, but not AC-T2, reflecting the transience of the stress response to AC (Figure 1). The oxidative stress response following exercise persisted for RE only, as seen by the continued activation of the glutathione pathway, e.g. 2-fold elevated cysteine-glutathione disulfide at RE-T2 (Figure 1). Mixed pro/anti-inflammatory signaling increased immediately following both exercises, as depicted by pro-inflammatory eicosanoids (12-HETE and 12-HHTrE) and the potent anti-inflammatory hormone corticosterone. This mixed pro/anti-inflammatory response returned to baseline for AC at T2, but continued for RE (Figure 5). Interestingly, several FA metabolites with anti-inflammatory and/or antioxidant properties (3-methyladipate, 16-hydroxypalmitate and the ω-3 PUFAs) were more elevated following AC than RE at both time points (Figure 2(a)). Together, these observations depict a more fleeting general stress response following AC than RE, even if initially more robust.

* Significantly different from resting (T0).

#### 5.2.4. Individual metabolites

Several metabolites showed evidence of an impactful change with exercise. As mentioned above, both exercise modalities brought about reduced levels of all eight acylcholines on the panel, including three phospholipids with polyunsaturated moieties, as well as glycerophosphoinositol (Figure 2(c)). Two polyunsaturated β-monoacylglycerides, 2-docosahexaenoylglycerol and the endocannabinoid 2-arachidonoylglycerol, were decreased by AC and RE at T1.

## 6. Discussion

Ultimately, both exercise modalities yielded largely similar metabolomic signatures. Hierarchical clustering and PCA both supported the idea of a largely common acute metabolomic response to aerobic and anaerobic modalities. Nevertheless, some unique individual metabolite and pathway differences were observed in the serum. As anticipated, both exercise sessions showed highly altered metabolomes immediately after exercise, but with a faster-than-expected regression toward baseline by an hour post-exercise. The degree of recovery was impressive, especially considering the strenuous nature of the exercise bouts used. Other studies report prolonged changes in post-exercise metabolite levels [8]. Most of those studies used sedentary, unhealthy, or diseased individuals. It is possible that a ‘healthy’ individual has a ‘healthy’ response characterized by the initial stress-response and a hasty return-to-baseline. In general, outside of the changeover to FA metabolism post-RE, this pattern was observed regardless of exercise mode or metabolic pathway.

Underlying disparities in intensity inevitably play out in energy dynamics and substrate selection, allowing for differences observed between AC and RE. AC was characterized by greater fuel flexibility during exercise, including markers of carbohydrate, amino acid, and FA oxidation, while the higher intensity of RE necessitated carbohydrate as a substrate. Higher lactate levels for RE than AC point to greater anaerobic glycolysis to meet the greater rate of ATP demand. Moreover, elevated blood-lactate coincided with limited FA metabolism during RE, potentially attenuating FA mobilization at RE-T1. By T2, both exercises displayed an FA-favored metabolic signature, coinciding with lactate’s return to baseline.

Differences in circulating tricarboxylic acid cycle metabolite levels may reflect differences in anaplerotic and cataplerotic activity associated with substrate choice surrounding each exercise bout as well. Citrate, which is generated before the α-ketoglutarate dehydrogenase step wherein most amino acids enter the tricarboxylic acid cycle, was greater for RE at T2, while succinate, which is generated after this step, was greater for AC at T1 and T2. Moreover, greater increases in argininosuccinate for AC-T1 suggested elevated urea cycling from heightened deamination during AC. Together, these observations point toward greater amino acid oxidation with AC. This was corroborated by larger decreases in circulating amino acid levels during AC than RE. Though assessment of serum, as opposed to cellular levels of these metabolites, provides only indirect evidence, elevated circulating intermediates have previously been reported to reflect cellular changes in response to both aerobic [5–14] and anaerobic [6,15–17] exercise. Future researchers are encouraged to use muscle biopsies to directly characterize intracellular changes.

Elevated purine turnover has been independently reported for both aerobic and anaerobic exercise [6,30] but never compared in the same cohort using a metabolomics platform. Higher values in purine salvage pathway metabolites observed for RE may reflect inherent differences between conditions. Repeated high-intensity exertions with intermittent rest periods during RE likely yielded large negative spikes in the ATP pool and greater disruptions in the purine salvage pathway. Xanthosine had the greatest average fold-changes in response to either exercise at either time point, exhibiting the potent impact of exercise seen throughout purine salvage metabolites. Elevated xanthosine could reflect greater tissue (muscle) damage incurred during RE. However, this seems unlikely, as other potential markers of muscle damage, 3-methyl-histidine, 2-aminioadipate and 5-methylthioadenosine, were only modestly changed (< 2-fold) and did not significantly differ between conditions. Notably, xanthine oxidase was not detected in the serum at any timepoint indicating xanthosine levels were increased from cellular release and not produced in circulation.

The current understanding of substrate selection during exercise reflects greater FA use with aerobic exercise [31]. As the largest potential energy depot in the body, fats, though slow to oxidize, provide the high-volume ATP production necessary during aerobic exercise. Ample evidence for greater FA oxidation during AC existed here. Various FA metabolites were 2-fold increased immediately following AC but not RE. Liver ketone body production increases in times of inadequate caloric availability [32]. Increased acetoacetate and β-hydroxybutyrate in response to AC, but not RE, suggest demand for substrate-level energy dynamics during AC exceeded intracellular provisions, potentially calling on delivery of ketones from hepatocytes, in response to the likely higher in-task caloric expenditure for AC than RE.

Heightened FA metabolism during AC came with changes across all forms: long-chain, medium-chain, PUFAs, amino-FAs, acylcarnitines, ketones, monohydroxyls, dicarboxylates, monoacylglycerides, lipid mediators, and membrane lipids (phospholipids, acylcholines, sphingolipids). Thus, many lipid metabolites mirrored those directly tied to substrate oxidation. In fact, perturbations in FA-derived signaling and effector molecules, like the eicosanoids, endocannabinoids, and their precursor PUFAs and β-monoacylglycerides, occurred at the same time as lipid metabolites linked to bioenergetics across exercise modes: elevated at AC-T1, AC-T2, and RE-T2. This *en masse* alteration of FA metabolites offers a potential mechanism for the expedited oxidative stress and inflammatory responses seen with AC. RE may rely more heavily on other endogenous, non-FA-derived antioxidant/inflammatory systems, thus delaying recovery. Alternatively, this may be an artifact of the specific metabolites on this panel. Regardless, if the lower initial rise in FA metabolism seen with RE does result in a greater oxidative and inflammatory stress and a stronger engagement of these other endogenous recovery systems, it could potentially result in more rapid adaptations with training. This may, in part, elucidate how higher intensity exercise leads to more rapid fitness gains.

Despite higher FA metabolism for AC at both times post-exercise, FA metabolites tended to increase from T1 to T2 for RE, but not AC. It has been suggested that while aerobic exercise uses more FA in-task, anaerobic exercise may lead to greater FA oxidation throughout the day [33]. Given the trend observed, it is not impossible that FA use at some time post-RE may have surpassed that of AC, but monitoring an extended post-exercise recovery period would be required to show this. Previous studies have reported post-exercise fat oxidation as a potential mechanism for fat mass changes following resistance training in men [34] and women [35]. Along those same lines, our findings indicate that despite the perception of a so-called fat burning zone during exercise, the post-exercise energy deficit appears to be recovered via FA oxidation regardless of exercise mode or fuel choice during exercise. Omega-3 FA supplementation has been purported to enhance sport performance [36] though mixed findings exist regarding their utility as an anti-inflammatory following resistance exercise [37,38]. Whether exogenous FA consumption is able to augment this process is not addressed here, but the observed increase in omega-3 FAs post-exercise offers the possibility.

Carbohydrate and AA metabolism were also elevated following both exercises, supporting the importance of protein in post-workout recovery [39,40]. While this is common practice amongst body-builders and strength athletes, even greater AA use during and following AC is potential evidence that endurance athletes may also benefit from protein intake in the peri-workout period. Metabolism was elevated through at least the hour post-exercise, for both RE and AC, even with ingestion limited to water-only for 2–3 hours prior to arrival and throughout testing. This may be particularly important when multiple efforts are required over the course of a day (e.g. rounds, tournaments, decathlon), as any nutritional practice to hasten recovery could be impactful.

### 6.1. Limitations

Participants and exercises used in this study well represent a healthy population’s response to real-world workouts done by active individuals. Further research is needed to examine diseased or clinical populations and different exercises. Still, the scope in terms of participant number (40) and metabolomic platform (754 metabolites) place this amongst the largest exercise metabolomics studies involving a repeated measures design to date. Reported supplement use included creatine and/or protein. It is not entirely impossible that these supplements could have influenced the panel. However, we believe discontinuance of a supplement could also have impacted results, and that our findings provide a template for comparison to a healthy cohort.

Nearly half of the available panel (355 of 754) consisted of lipid metabolites. As a result, analytical techniques may have been biased toward concluding AC had a larger effect than RE. A predominance of lipids within metabolomic panels is common, and metabolite selection should be considered in panel design, analysis, and interpretation.

Despite substantial shifts in blood flow during exercise routing the majority of cardiac output toward skeletal muscle, it remains impossible to determine which tissues were involved in the uptake and release of measured metabolites. Still, the selection of serum rather than muscle tissue as a biological medium may better reflect systemic-level metabolic activity and substrate mobilization, but only indirectly captures intramuscular metabolism. For example, extra-muscular adipocytes may have played a larger role than intramuscular triglycerides in determining the ‘lipidomic’ profiles observed. Use of different tissue or carefully timed arterial-plus-venous sampling may offer more insight.

Finally, time-point selection offered insight into the acute metabolomic response yet did not assess fleeting changes inside of the 1-hour recovery period, nor delayed responses beyond this time frame, including metabolites requiring more time for gene expression and downstream production. Investigations using various time points are needed.

## 7. Conclusions

Exercise requires energy, and the form of energy used shapes the metabolomic signature of the exercise bout. In turn, it is the type of exercise that determines the form of energy. AC yielded greater substrate catabolism with high fuel flexibility during exercise. In task, AC relied on ‘sustainable’ energy in the form of FA and aerobic oxidization of substrates. The resulting global lipidomic response included downstream lipid mediators. With roles as signaling and effector molecules within processes like anti-oxidation and the pro/anti-inflammatory responses, increases in these metabolites may have expedited recovery. In contrast, RE showed greater elevations in glycolysis and purine salvage metabolites to meet the higher ATP-turnover rate required during such high-intensity exercise. Post-exercise, FA metabolism appeared to be the main contributor to bioenergetic need. Although aspects of recovery were seen within the 1-hour time course, the anaerobic energy deficit from RE led to a comparatively protracted recovery, as noted by the slower pro-to-anti-inflammatory shift during recovery, and continued rise in purine salvage and FA metabolites.

Although it is tempting to fixate on differences between exercises, the degree of similarity in response to AC and RE was striking. Unlike what has been noted for enzyme activity [16,29], directional differences were absent. Not a single metabolite increased in response to one exercise and decreased in response to the other. Generally, greater perturbations to the resting metabolome were elicited by AC, though further study is needed to determine if that reflects differences in total workload or intensity. The disparity largely included lipidomic changes. Both exercise metabolomes were predominantly characterized by the activation of various bioenergetic systems and the stress-response. Overall, beyond magnitude and time course, differences between AC and RE were fairly limited, and aerobic and anaerobic exercise seem to have far more similarities than differences, suggesting a fairly robust exercise response.

Several additions to the exercise metabolomics literature are offered. This is the first direct comparison of serum metabolomic responses to aerobic and anaerobic exercise bouts in a single cohort of healthy human participants using a crossover design. Exercise protocols employed in metabolomics research are often dissimilar to those used by actual exercisers and athletes. Data on the acute response to resistance training without a concurrent nutritional intervention are particularly lacking. The current study addresses these issues in the context of comparing and contrasting the aerobic and anaerobic exercise responses immediately and 1-hour into the post-exercise recovery period.

## Supplementary Material

Supplemental Material

Supplemental Material

Supplemental Material

Supplemental Material
