# Supplementary material for: The exercise metabolome: acute aerobic and anaerobic signatures
Source: J Int Soc Sports Nutr. 2022 Oct 11;19(1):603–22. doi: 10.1080/15502783.2022.2115858 (PMC9559054; doi:10.1080/15502783.2022.2115858)
Supplement: Supplemental Material [file RSSN_A_2115858_SM1354.pdf]

Super Pathway	Sub Pathway	Metabolite	AC-T1	RE-T1	AC-T2	RE-T2
Nucleotide	Purine , (Hypo)Xanthine/Inosine	xanthosine * **	**6.40**	**12.49**	**4.83**	**13.70**
		hypoxanthine* **	**2.40**	**4.95**	**1.44**	**2.95**
Carbohydrate	Glucose	lactate *	**3.40**	**4.44**	1.48	1.74
		pyruvate	3.28	3.57	1.39	1.68
Vitamins	Pantothenate	pantothenate *	**1.78**	**2.36**	1.25	1.44
Energy	Oxidative Phosphorylation	phosphate * **	**2.12**	**1.25**	**1.08**	**0.44**
	TCAC	malate	4.09	4.05	1.67	1.83
		fumarate	3.58	3.19	1.55	1.42
		alpha-ketoglutarate	2.36	2.30	1.42	1.45
		succinate * **	**4.21**	**1.78**	**1.60**	**1.01**
		citrate **	1.39	1.79	**1.58**	**2.15**
	TCAC & Glutamate	beta-citrylglutamate * **	**2.56**	**1.89**	**1.30**	**0.97**
Amino Acid	Glutathione	cysteinylglycine	3.11	3.10	1.83	1.88
		cysteine-glutathione disulfide	1.67	1.31	1.45	2.01
	Glutathione & Energy	alpha-ketobutyrate	1.79	1.46	2.38	2.30
		2-hydroxy(iso)butyrate *	**1.75**	**2.04**	2.34	2.37
	Methionine & Cysteine	cysteine sulfinic acid *	**0.68**	**0.48**	0.87	0.85
		N-acetylmethionine	2.21	2.12	1.53	1.61
	Phenylalanine and Tyrosine	3-(3-hydroxyphenyl)propionate sulfate *	**2.41**	**1.41**	1.09	0.89
		4-hydroxyphenylpyruvate *	**3.36**	**2.87**	1.31	1.32
		vanillylmandelate (VMA) * **	**2.66**	**1.55**	**1.89**	**1.33**
		vanillic alcohol sulfate	0.61	0.62	0.30	0.37
		3-(4-hydroxyphenyl)lactate	1.68	1.83	1.68	2.00
	Tryptophan	N-acetyltryptophan	2.12	1.60	1.01	1.11
		xanthurenate * **	**2.08**	**1.21**	**1.41**	**0.93**
	Urea cycle & Arginine	argininosuccinate	2.34	2.00	1.57	1.31
	BCAA	2,3-dihydroxy-2-methylbutyrate	1.71	1.84	2.09	2.37
		alpha-hydroxyisocaproate *	**1.92**	**2.36**	1.53	1.90
		3-hydroxyisobutyrate	1.92	1.93	2.02	2.08

Super Pathway	Sub Pathway	Metabolite	AC-T1	RE-T1	AC-T2	RE-T2
Lipid	FA (BCAA)	propionylcarnitine (C3) * **	2.43	1.20	1.23	0.97
	Ketone Bodies	acetoacetate * **	2.10	0.90	2.92	1.29
		3-hydroxybutyrate (BHBA) * **	2.43	1.09	3.47	1.45
	FA (Carnitine)	3-hydroxybutyrylcarnitine (1)	2.75	2.17	2.98	2.10
		3-hydroxybutyrylcarnitine (2)	3.23	2.72	2.56	1.90
		acetylcarnitine (C2)	2.88	2.47	1.99	1.78
		propionylcarnitine (C3) * **	2.43	1.20	1.23	0.97
		decanoylcarnitine (C10) *	1.03	0.46	0.89	0.79
		suberoylcarnitine (C8-DC) **	1.58	1.27	2.37	1.19
	FA (Glutamine)	hexanoylglutamine **	1.84	1.35	2.72	1.32
	FA Synthesis	malonate *	1.61	2.24	1.89	2.71
	Steroid	corticosterone **	3.43	3.21	1.47	2.07
	Eicosanoid	12-HHTrE **	2.35	2.33	1.83	2.23
		12-HETE **	3.07	2.77	1.76	2.57
	FA, Dicarboxylate	azelate (nonanedioate) *	1.64	3.05	0.81	2.23
		maleate	1.84	2.00	1.56	1.63
		hexadecanedioate * **	1.86	1.07	2.21	1.08
		undecanedioate	1.55	2.01	0.74	1.46
		tetradecanedioate *	1.66	0.93	2.46	1.43
		3-methyladipate *	2.60	1.88	1.87	1.26
	FA, Monohydroxy	3-hydroxysebacate * **	2.09	1.56	3.07	1.39
		9-hydroxystearate *	2.29	1.07	1.92	1.70
		alpha-hydroxycaproate *	1.93	2.25	1.49	1.74
		16-hydroxypalmitate *	2.37	1.08	2.11	1.61
	Long Chain FA	myristate (14:0) *	2.80	1.14	2.03	1.81
		margarate (17:0) *	2.03	1.05	1.84	1.43
		palmitate (16:0) *	2.01	1.01	1.70	1.44
		palmitoleate (16:1n7) *	3.31	1.07	2.43	2.43
		myristoleate (14:1n5) *	2.61	0.96	2.21	2.14
		10-heptadecenoate (17:1n7) *	3.09	1.08	2.37	1.91
		eicosenoate (20:1) * **	2.29	1.01	2.27	1.57
		10-nonadecenoate (19:1n9) *	2.36	1.22	2.22	1.62
		oleate/vaccenate (18:1) *	2.41	1.07	2.02	1.60
	Medium Chain FA	5-dodecenoate (12:1n7) *	2.13	0.91	1.79	1.89
		laurate (12:0) *	2.47	0.98	1.90	1.64
	Monoacylglycerol	2-docosahexaenoylglycerol (22:6)	0.49	0.35	0.82	0.98
		2-arachidonoylglycerol (20:4)	0.50	0.46	0.92	1.03
	Phospholipid	glycerophosphoinositol	0.36	0.28	0.83	0.73
		dihomo-linolenoyl-choline	0.31	0.29	0.84	0.93
		arachidonoylcholine	0.35	0.31	0.79	0.93
		docosahexaenoylcholine	0.36	0.31	0.79	0.90

Super Pathway	Sub Pathway	Metabolite	AC-T1	RE-T1	AC-T2	RE-T2
	Polyunsaturated FA (n3 and n6)	linolenate [α/γ (18:3n3 or 6)] *	**3.31**	**1.01**	2.40	1.79
		stearidonate (18:4n3) *	**3.00**	**1.20**	2.27	1.75
		docosatrienoate (22:3n3) *	**2.65**	**1.11**	2.25	1.73
		dihomo-linoleate (20:2n6) *	**2.06**	**1.11**	1.99	1.61
		linoleate (18:2n6) *	**2.33**	**0.94**	1.88	1.50
	FA (Choline)	palmitoloelycholine **	0.33	0.45	**0.78**	**0.96**
		stearoylcholine **	0.29	0.26	**0.78**	**0.92**
		palmitoylcholine **	0.32	0.30	**0.78**	**0.89**
		oleoylcholine **	0.31	0.30	**0.77**	**0.91**
		linoleoylcholine	0.32	0.29	0.75	0.86
	Sphingolipid	sphingosine	2.09	1.76	1.33	1.25
	Primary Bile Acid	glycocholate	0.55	0.46	0.49	0.37
		taurocholate *	**0.73**	**0.47**	0.43	0.42
		glycochenodeoxycholate	0.52	0.47	0.59	0.54
		tauro-beta-muricholate	0.54	0.48	0.54	0.51
		cholate *	**0.56**	**0.86**	0.45	0.47
	Secondary Bile Acid	glycohyocholate	0.43	0.42	0.59	0.42
		glycoursodeoxycholate	0.44	0.43	0.62	0.58
		glycodeoxycholate	0.49	0.51	0.48	0.46
		tauroursodeoxycholate * **	**0.68**	**0.39**	**0.61**	**0.92**
		taurodeoxycholate	0.62	0.51	0.41	0.46
		ursodeoxycholate	0.63	0.67	0.48	0.53
Xenobiotics	Food Component/Plant	ferulic acid 4-sulfate *	**1.17**	**0.81**	0.64	0.43
		4-vinylguaiacol sulfate	0.77	0.56	0.35	0.33
		eugenol sulfate	0.78	0.75	0.50	0.59
