# Supplementary figures and images for: The exercise metabolome: acute aerobic and anaerobic signatures
Source: J Int Soc Sports Nutr. 2022 Oct 11;19(1):603–22. doi: 10.1080/15502783.2022.2115858 (PMC9559054; doi:10.1080/15502783.2022.2115858)

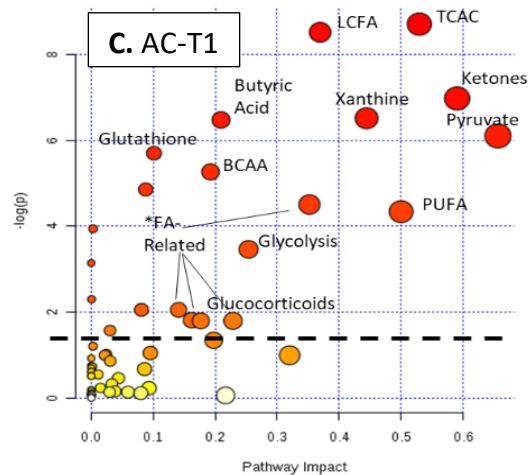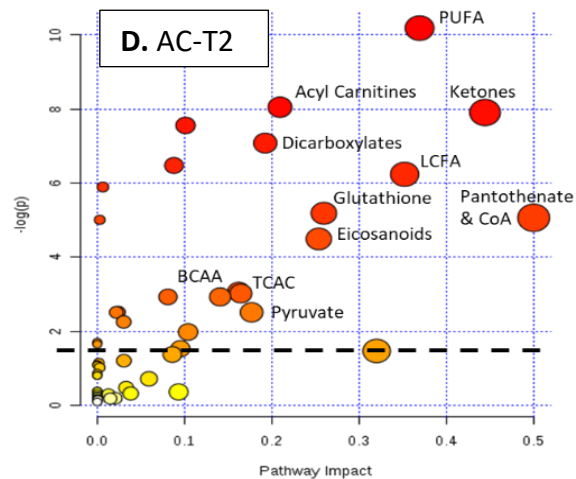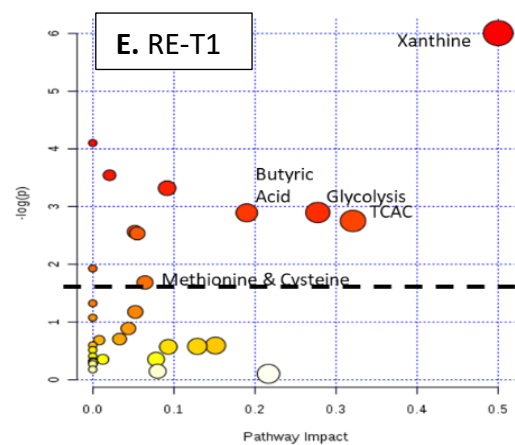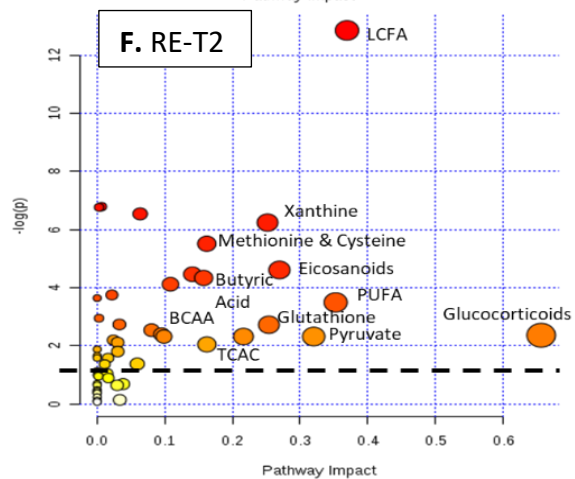

Supplement: Supplemental Material [file RSSN_A_2115858_SM1345.pdf]

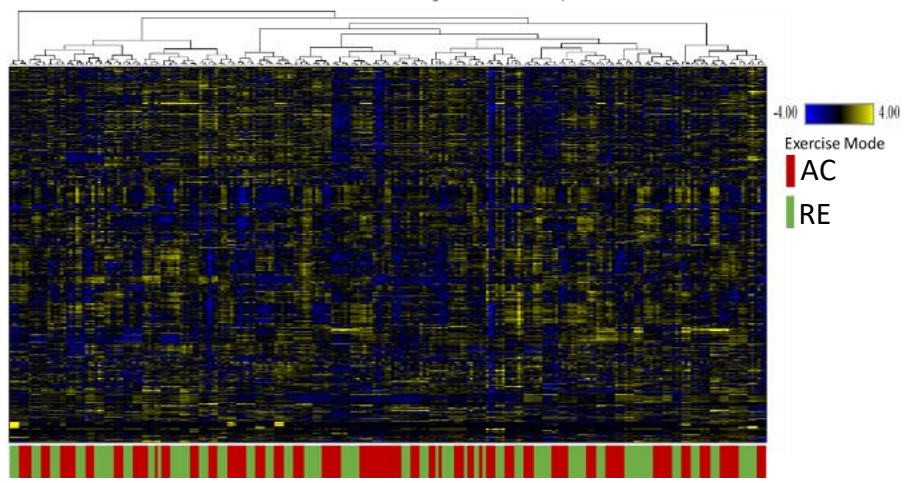

Supplement: Supplemental Material [file RSSN_A_2115858_SM1339.pdf]

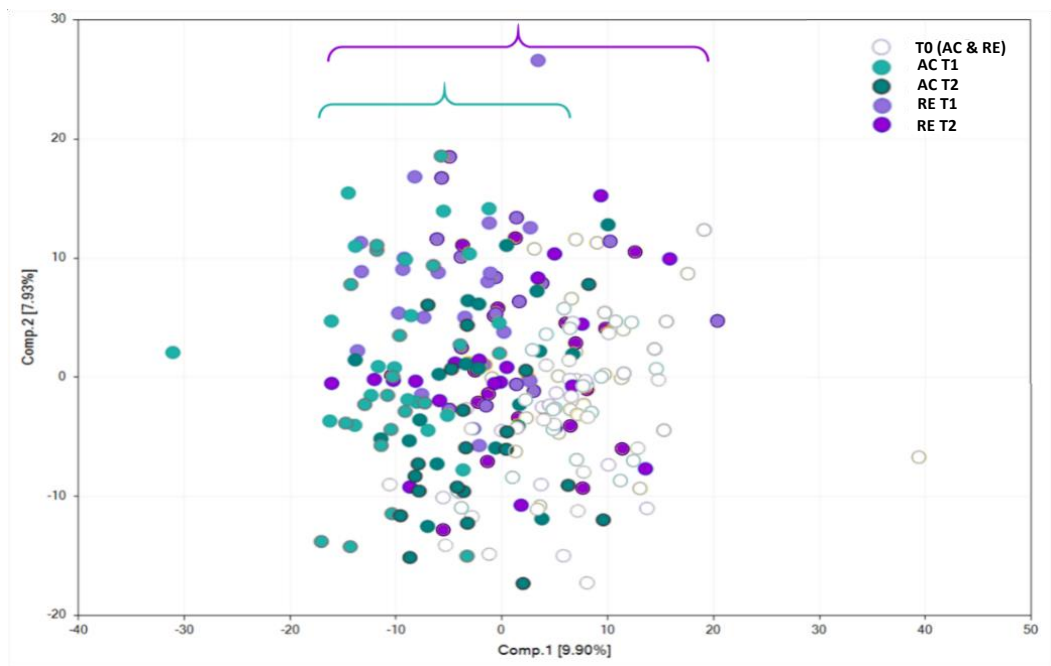

Supplement: Supplemental Material [file RSSN_A_2115858_SM1335.pdf]
